# Two Distinct Moral Mechanisms for Ascribing and Denying Intentionality

**DOI:** 10.1038/srep17390

**Published:** 2015-12-04

**Authors:** Lawrence Ngo, Meagan Kelly, Christopher G. Coutlee, R. McKell Carter, Walter Sinnott-Armstrong, Scott A. Huettel

**Affiliations:** 1 Medical Scientist Training Program, Duke University School of Medicine Box 102005 DUMC, Durham, NC 27710; 2 Department of Neurobiology, Duke University School of Medicine 412 Research Drive, Box 3209, Durham, NC 27710; 3 Kenan Institute for Ethics, Duke University Box 90432, Durham, NC 27708; 4 Program in Neurosciences, Duke University Levine Science Research Center, Durham, NC 27710; 5 Department of Psychology and Neuroscience, Duke University Box 90086, 417 Chapel Drive, Durham, NC 27710; 6 Center for Cognitive Neuroscience, Duke University Box 90999, Durham, NC 27710; 7 Brain Imaging Analysis Center, Duke University Medical Center 2424 Erwin Road, Suite 501, Durham, NC 27705; 8 Institute of Cognitive Science, University of Colorado Boulder 1777 Exposition Dr., Room 171, Boulder, CO, 80301; 9 Department of Psychology and Neuroscience, University of Colorado Boulder Muenzinger Psych Building, UCB 344, Boulder, CO, 80309; 10 Department of Philosophy, Duke University 201 West Duke Building, Box 90743, Durham, NC 27708.

## Abstract

Philosophers and legal scholars have long theorized about how intentionality serves as a critical input for morality and culpability, but the emerging field of experimental philosophy has revealed a puzzling asymmetry. People judge actions leading to negative consequences as being more intentional than those leading to positive ones. The implications of this asymmetry remain unclear because there is no consensus regarding the underlying mechanism. Based on converging behavioral and neural evidence, we demonstrate that there is no single underlying mechanism. Instead, two distinct mechanisms together generate the asymmetry. Emotion drives ascriptions of intentionality for negative consequences, while the consideration of statistical norms leads to the denial of intentionality for positive consequences. We employ this novel two-mechanism model to illustrate that morality can paradoxically shape judgments of intentionality. This is consequential for *mens rea* in legal practice and arguments in moral philosophy pertaining to terror bombing, abortion, and euthanasia among others.

Intentionality is foundational to many of our social interactions and institutions. Traditionally in the law, it escalates a lesser charge of manslaughter to first-degree murder^1^. In the recent case of *Rosemond v. United States*, intentionality’s role in determining culpability for *aiding and abetting* was decided by the US Supreme Court^2^. In addition, a long lineage of moral theories originating from Aquinas’s *Doctrine of Double Effect* from the Middle Ages^3^ to contemporary theories of a universal moral grammar^4^ hold that intentionality serves solely as an input for moral judgments, rather than the reverse.

These traditional assumptions seem to be challenged by the following vignette from the nascent field of experimental philosophy^5^,^6^,^7^:

The CEO knew the plan would *harm* the environment, but he did not care at all about the effect the plan would have on the environment. He started the plan solely to increase profits. Did the CEO intentionally *harm* the environment?

Most participants say “yes”^8^,^9^,^10^,^11^. But now change a single word:

The CEO knew the plan would *help* the environment, but he did not care at all about the effect the plan would have on the environment. He started the plan solely to increase profits. Did the CEO intentionally *help* the environment?

Here, most participants say “no”^8^,^9^,^10^,^11^. Through vignettes like these, experimental philosophers have repeatedly shown that actions leading to negative consequences are judged as being more intentional than otherwise similar actions leading to positive consequences—often called the *Knobe Effect* (*KE*)^9^,^10^,^11^.

There is controversy over whether the *KE* truly represents a violation of the assumptions of traditional moral and legal theory^10^,^12^,^13^,^14^,^15^,^16^,^17^,^18^,^19^. In these domains, judgments of intentionality are assumed to be inputs for moral judgments (e.g., the act of intentional killing is held to be more morally wrong than the act of accidental killing). However, the *KE* demonstrates a case in which moral judgments can serve as inputs into judgments of intentionality, which raises the possibility of circularity in legal and moral frameworks (e.g., people who commit heinous crimes might be more likely to be seen as intentionally acting, thus leading to different charges).

Elucidating the mechanisms of the *KE* is integral to resolving this controversy. Several theories have been proposed to explain the mechanism behind the KE. The emotional theory (also called the motivational bias theory) holds that negative consequences elicit emotions that make participants want to blame the agent, and therefore, drive up ratings of intentionality. For positive consequences, such negative emotions do not arise, so participants’ ascriptions of low intentionality remain unbiased^10^,^15^. Alternatively, the conversational pragmatics theory proposes that the *KE* is a linguistic rather than an emotional process. The KE exists because participants use the relevant conversational context to predict what the questioner really wants to know and respond accordingly^20^. Despite each of these theories’ applicability to some empirical data, no consensus yet supports any single theory or mechanism for the *KE*^10^.

One promising approach to elucidating the *KE*’s mechanism has been examining related individual-difference measures. A previous study demonstrated a correlation of the *KE* with performance on the Cognitive Reflection Task (CRT), which is a measure of how well participants can suppress spontaneous responses, so-called *system 1* responses, in favor of more deliberative, *system 2* responses^21^,^22^. Participants scoring higher on the CRT exhibited less of a *KE*, suggesting that the *KE* may arise from a *system 1* process. Another study showed an association between the *KE* and the extraversion subscale of the Big Five model of personality, such that more extraverted participants exhibited more of a *KE*. The authors speculated that since extraversion is correlated with emotional expressiveness, the correlation serves as support for the emotional theory^23^. In Experiment 1, we sought to replicate these findings, as well as to survey a broad range of individual-difference measures drawn from personality psychology, decision making, and moral psychology (Supplementary Methods), whose potential correlations with the *KE* could have varying implications for theories of mechanism. We created a set of 15 novel scenarios—each with a negative and positive consequence variant—modeled after the original vignettes^8^. Participants responded on a scale from 1 (Not Intentionally at All) to 8 (Completely Intentionally) in a self-paced task allowing for the measurement of response times.

Considering the extensive connections in the literature made between emotional processing and moral judgments^24^,^25^,^26^,^27^, we sought in Experiment 2 to specifically test the one-mechanism hypothesis that emotional salience alone accounts for the asymmetry: negative consequences are judged as more intentional than positive consequences because negative consequences are more emotionally salient. We presented participants from the online labor market Amazon Mechanical Turk (MTurk) with the original negative and positive conditions of the *KE*^8^. We also included a novel low-salience, neutral condition (Supplementary Methods) that the emotional salience model would predict to have the lowest intentionality rating of all.

In Experiment 3, we conducted a neuroimaging experiment intended to provide converging behavioral and neural evidence regarding the mechanism of the *KE*. A significant portion of the neuroscience of morality has focused on the role of regions classically associated with emotion and theory of mind processing. For emotion, a wide network of regions has been implicated including the upper midbrain, orbital and medial prefrontal cortex, superior temporal sulcus, and amygdala^24^. Alternatively, a different network of regions has been implicated for theory of mind, including the ventromedial prefrontal cortex (VMPFC), temporoparietal junction (TPJ), and precuneus/posterior cingulate^28^,^29^,^30^. In relation to the intersection between emotion and theory of mind, one model holds that *affective theory of mind*, an entity that is dissociable from *cognitive theory of mind*, employs faster and more automatic circuits involving the amygdala^31^. We leveraged this prior work concerning neural processing of emotion and theory of mind to help test a two-mechanism model for the asymmetry: emotion drives ascriptions of intentionality for negative consequences, while the denial of intentionality for positive consequences depends on the consideration of statistical norms (i.e., how common certain actions are perceived to be in the general population). Participants were presented with the scenarios from Experiment 1 — along with 25 novel scenarios — in an fMRI scanner (Supplementary Methods; Fig. 1a). In a post-scan session, participants were asked to rate the same vignettes regarding three other factors: *emotional reaction*, *statistical normativity*, and *moral judgment*.

## Results

### Experiment 1

**Figure 1:**
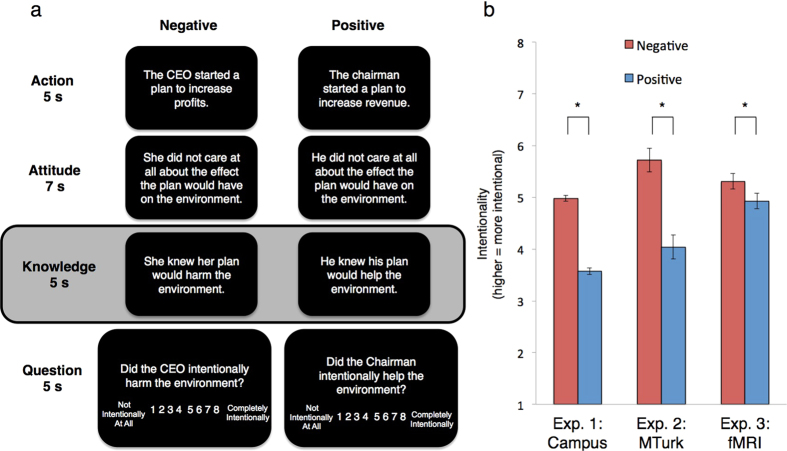
Asymmetries in intentionality are robust across three different methods of experimentation. (**a**) In the fMRI version of the task, participants read and responded to two versions of each general story. These versions differed in whether the agent’s actions lead to morally negative or positive consequences (40 pairs for 80 vignettes total). Participants provided ratings of intentionality on a scale from 1 (Not Intentionally at All) to 8 (Completely intentionally), and the direction of the scale was counterbalanced trial-by-trial. Reported imaging results are derived from data collected during the “Knowledge” epoch. The ITI was 2 s. (**b**) At the group level, participants consistently rated actions in negative conditions as being more intentional than those in positive conditions across three different experiments. Model-free means are presented along with 95% confidence intervals for comparison across three different experimental designs. *Indicates that the means are different according to paired *t*-tests.

We replicated the *KE* in our novel set of scenarios: intentionality ratings for negative conditions were higher than those for positive conditions (paired *t*(2543) = 28.3, *p* < 0.0001; Fig. 1), which is consistent with previous findings^8^,^9^,^10^,^11^. A hierarchical, mixed-effects model with valence and trial number as fixed effects and participant as a random effect confirmed a main effect of valence (*β* = 1.41, *t*(282) = 36.4, *p* < 0.0001). Given the repetition and length of our task, we also found evidence for practice effects: there was a significant valence × trial number interaction (*β* = −0.01, *t*(8049) = −3.15, *p* = 0.005) such that intentionality ratings for positive conditions significantly increased over successive trials while those for negative conditions significantly decreased (Supplementary Table 1). In a separate but analogous model with response time as the dependent variable, we found significantly longer decision times for positive conditions compared to those for negative conditions (*β* = −0.07, *t*(282) = −7.07, *p* < 0.0001; Supplementary Table 2).

We found no significant correlations between any of the individual-difference scales or their component sub-scales with participants’ mean differences between ratings for negative and positive consequences. In exploratory analyses not corrected for multiple comparisons, there was a positive correlation between ratings for negative consequences and measures of moral harm sensitivity on the Moral Foundations Questionnaire^32^, and this was replicated across two rounds of experimentation (*r* = 0.23, *p* = 0.05, *N* = 68 and *r* = 0.30, *p* = 0.01, *N* = 70)—making it a target for future studies. Overall, the lack of any strong correlations between the *KE* and a broad battery of individual-difference measures was consistent with the hypothesis that there exists no single underlying mechanism for the *KE*.

### Experiment 2

We again replicated the *KE* such that intentionality ratings for negative conditions were higher than those for positive conditions (paired *t*(385) = 11.3, *p* < 0.0001; Fig. 1). We found salience ratings to be higher for negative conditions than for positive conditions (Supplementary Fig. 1; paired *t*(385) = 2.28, *p* = 0.02). However, the low-salience (neutral) condition had higher ratings of intentionality compared to the positive condition (paired t(385) = 2.58, p < 0.01; Supplementary Fig. 1) even though salience ratings were much lower for the former compared to the latter (paired *t*(385) = 17.8, *p* < 0.0001; Supplementary Fig. 1b). When analyzing the different condition valences independently, we found an effect of emotional salience in predicting ratings of intentionality only in the negative condition (*β* = 0.12, *t*(384) = 3.24, *p* = 0.001), but not for neutral (*β* = 0.02, *t*(384) = 0.47, *p* = 0.64) or positive conditions (*β* = 0.04, *t*(384) = 0.93, *p* = 0.35; Supplementary Fig. 1c–e).

### Experiment 3

**Figure 2:**
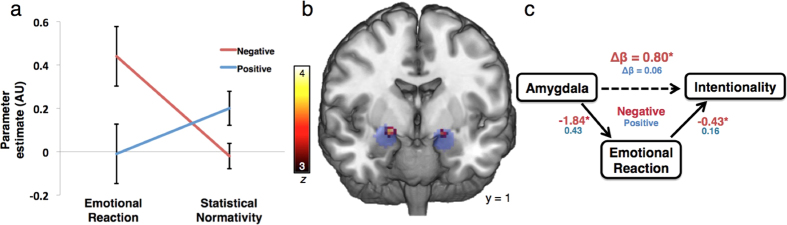
Converging behavioral and neural evidence suggests that *Ascription* leads to higher intentionality through an emotional mechanism while *Denial* leads to lower intentionality and is dependent on *statistical normativity*. (**a**) Behaviorally, *emotional reaction* significantly predicts intentionality ratings for negative conditions but not for positive conditions. Conversely, *statistical normativity* predicts intentionality ratings for positive conditions but not for negative conditions. The parameter estimates and 95% confidence intervals are presented from the hierarchical, mixed-effects model. (**b**) Activation in bilateral dorsal amygdala (red-yellow colormap) was found to be positively associated with intentionality ratings for negative outcomes within ROIs identified from reverse inference maps of “emotion” from Neurosynth, indicated in blue^66^,^67^. (**c**) This relationship was partially mediated by reports of emotion for negative consequences (Indirect Effect Estimate (Δ*β*) = 0.80; 95% confidence interval = [0.07, 2.02]) while reports of positive emotion did not have a mediating role (Supplementary Table 4). *Emotional reaction* ratings are presented on a valenced scale, such that negative values indicate stronger negative emotional responses. *β* for separate negative and positive consequence mediation models are indicated, while the Δ*β* indicates the change in beta value for the direct path after controlling for the indirect path.

We replicated in a third sample the asymmetry such that intentionality ratings for negative conditions were higher than those for positive conditions (paired *t*(593) = 3.79, *p* = 0.002; Fig. 1). However, in a hierarchical, mixed-effects model that included *emotional reaction* and *statistical normativity*, we found no main effect of condition valence (*β* = 0.12, *t*(15) = 0.82, *p* = 0.43; Supplementary Table 3). Furthermore, the regressors revealed a double dissociation in the mechanisms for negative and positive conditions: there was a significant valence × emotional reaction interaction (*β* = 0.45, *t*(1209) = 4.32, *p* < 0.0001) as well as a significant valence × statistical normativity interaction (*β* = −0.23, *t*(1209) = −4.59, *p* < 0.0001). Interrogation of these interactions yielded a significant effect for emotional reaction in negative (*β* = 0.44, *t*(1209) = 6.30, p < 0.0001) but not in positive conditions (*β* = -0.01, *t*(1209) = −0.16, *p* = 0.87) and a significant effect for statistical normativity in positive (*β* = 0.20, *t*(1209) = 5.39, *p* < 0.0001) but not in negative conditions (*β* = −0.03, *t*(1209) = −0.80, *p* = 0.42; Fig. 2a; Supplementary Table 3). More negative emotional reaction ratings predicted higher intentionality ratings in negative conditions while smaller appraisals of statistical normativity (more rare) predicted lower intentionality ratings in positive conditions (Supplementary Table 3).

For our neuroimaging analyses, we first defined regions of interest in bilateral dorsal amygdala based on the reverse-inference map for the term “emotion” from Neurosynth (Fig. 2b). The fMRI response extracted from these peaks supported a significant mediation model that held that higher individual activation in the amygdala led to more negative *emotional reactions,* which in turn led to higher ratings of intentionality (Fig. 2c, Supplementary Table 4).

A control mediation analysis using amygdala data drawn from positive conditions was not significant (Fig. 2c, Supplementary Table 4), supporting the inference that processing in the amygdala specifically supports judgments of intentionality for negative outcomes. A whole-brain search for regions associated with intentionality in negative conditions identified a cluster in the left dorsolateral prefrontal cortex (Supplementary Fig. 2). As another negative control, we also failed to find a significant mediation model for signal extracted from DLPFC and ratings of intentionality by *emotional reaction* (Supplementary Table 4).

A direct contrast of positive >negative conditions revealed heightened activation in a distributed pattern of brain regions including lateral prefrontal cortex (Supplementary Fig. 2, Supplementary Table 5). The direct contrast of negative >positive conditions did not yield any significant areas of activation. Additionally, we replicate the significant vignette valence × trial number intentionality rating interaction (*β* = −0.01, *t*(1209) = −2.31, *p* = 0.02) from Experiment 1.

**Figure 3:**
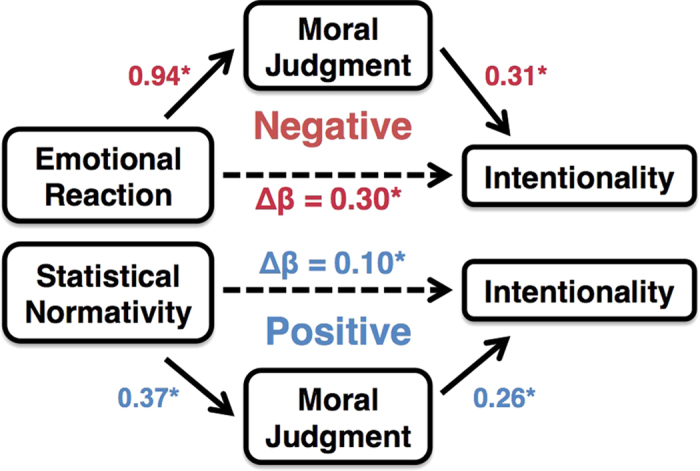
Moral judgments of blame and credit serve as inputs for intentionality ascription in both *Ascription* and *Denial*. *Moral judgment* of blame served as a significant mediator of the relationship between *emotional reaction* and *intentionality* in negative conditions (Indirect Effect Estimate (Δ*β*) = 0.30; 95% confidence interval = [0.18, 0.43]). *Moral judgment* of credit served as a significant mediator of the relationship between *statistical normativity* and *intentionality* in positive conditions (Indirect Effect Estimate (Δ*β*) = 0.10; 95% confidence interval = [0.05, 0.13]; Supplementary Table 6).

Finally, in our mediation models of whether moral judgments can serve as inputs for judgments of intentionality vs. the reverse, we found *moral judgment* of blame to significantly mediate the relationship between *emotional reaction* and *intentionality* for negative conditions (Indirect Effect Estimate (Δ*β*) = 0.30; 95% confidence interval = [0.18, 0.43]) and *moral judgment* of credit to significantly mediate the relationship between *statistical normativity* and *intentionality* for positive conditions (Indirect Effect Estimate (Δ*β*) = 0.10; 95% confidence interval = [0.05, 0.13]; Fig. 3, Supplementary Table 6). Though significant, the models using *intentionality* as the mediator yielded significantly smaller mediating effects for both conditions (Supplementary Table 6).

## Discussion

Across a series of three experiments, converging behavioral and neural evidence demonstrates two distinct and dissociable mechanisms for judgments of intentionality. Emotion drives higher ascriptions of intentionality for negative consequences, while statistical norms derived from beliefs about how often people behave in similar ways underlie the denial of intentionality for positive consequences. Further analysis shows that moral judgments of blame and credit can serve as inputs for intentionality judgments, rather than only the other way around.

In Experiment 1, we searched broadly for individual-difference measures correlated with the *KE*, but could not find any strong associations across several rounds of experimentation. This includes a failure to replicate previous associations that have been found between the *KE* and the Cognitive Reflection Task (CRT) and extraversion subscale of the Big Five Personality Inventory (NEO PI-R)^12^,^21^. We did find, in both samples, a tentative correlation between the *KE* and the Moral Foundations Questionnaire’s (MFQ’s) Harm subscale^33^. The MFQ evaluates sensitivity to various psychological systems that are held to be the foundation of “intuitive ethics,” which includes care/harm, fairness/cheating, and authority/subversion, among others^33^,^34^. Interestingly, all the negative consequences in our set of scenarios have to do with harm rather than any of the other moral foundations, consistent with the sort of specificity that the Moral Foundations Theory would predict. Because this effect was identified in *post hoc* analyses and was not originally posited, future research will be necessary for confirmation.

In experiment 2, we demonstrated that a one-mechanism, emotional-salience model was not a complete explanation for the *KE*. There was no close mapping of salience to intentionality across the negative, neutral, and positive conditions. While ratings of intentionality exhibited a negative, linear trend across these three conditions, the ratings of salience, instead, exhibited a u-shaped curve across the three conditions (Supplementary Figure 1a-b) with ratings of salience for the neutral condition near floor. Although the emotional salience model could not explain behavior across all conditions, we demonstrated that the emotional salience model was specifically predictive for intentionality ratings for negative conditions. These results lend further support to the claim that two mechanisms may be responsible for giving rise to the asymmetry. However, there were still challenges to this interpretation: self-reports of emotional salience may not be entirely reliable, and the mechanism underlying positive consequences remains unknown. Further, even though the role of emotion has been implicated, it is unclear what role morality judgments have in influencing intentionality judgments. Experiment 3 was designed to address all of these issues using converging behavioral and neural evidence.

In Experiment 3, we demonstrated three major results. First, there was a behavioral double-dissociation between *emotional reaction* and *statistical normativity* in predicting ratings of intentionality for negative and positive conditions, respectively. Next, we found higher levels of activation in the amygdala were associated with higher ratings of intentionality for negative conditions, and this relationship was mediated by ratings of *emotional reaction*. Finally, we found that judgments of moral blame or credit mediated the relationship between *emotional reaction* and *statistical normativity* in negative and positive conditions, respectively.

Regarding the first of these results, we found that the addition of *emotional reaction* and *statistical normativity* to our behavioral model made the main effect of valence diminish to non-significance (as compared to the simpler model from experiment 1). This is consistent with the fact that these new variables explain much of the variance associated with the *KE* asymmetry.

In the neuroimaging analysis, we identified the amygdala as the region of the brain that had the highest *z-score* for a reverse inference on the term “*emotion*.” Though other brain regions normally implicated in emotion such as the ventromedial prefrontal cortex, insula, medial orbitofrontal cortex, and anterior cingulate cortex all also had relatively high *z-scores*, we chose to limit our analyses to the amygdala for two reasons. First, even among these high-scoring regions, the amygdala’s selectivity was an outlier compared to the rest of the brain, more than doubling the *z-score* of any other single region. Second, our main priority was using the neural data as a marker for whether emotion was involved in the *KE*. Further studies analyzing these other areas of emotional processing may yield more complete results regarding the neural circuitry underlying the *KE*. However, the more narrow scope of the present analyses was designed to mitigate problems both with multiple comparisons and with reverse inference. In this attempt, we limited our analyses to only the main result and those pertaining to the two negative controls designed to address potential confounds in mediation analyses^35^.

In one of these analyses, a whole-brain analysis demonstrated an association between DLPFC activation and intentionality ratings for negative conditions. This finding is consistent with a previous literature distinguishing between cognitive and affective theory of mind (ToM)^31^,^36^. While cognitive ToM aims to interpret another’s knowledge, affective ToM interprets another’s internal emotions. Repeated transcranial magnetic stimulation to the DLPFC has been shown to induce a selective effect on cognitive but not affective ToM^31^. Our study demonstrates a distinct yet complementary dissociation, where *emotional reaction* mediated the amygdala’s association with intentionality ratings but did not do so for the DLPFC.

Several ancillary findings from Experiment 3 converged with results found in Experiment 1. The distributed network of activation for the positive > negative main effect contrast (Supplementary Fig. 2) is consistent with longer participant reaction times for positive compared to negative consequences in Experiment 1. The lack of significant areas for the negative > positive contrast may be related to the diminished effect size of the behavioral *KE* that was observed in Experiment 3 in comparison to the other two experiments (Fig. 1). We found that this was in part due to an accentuation in the magnitude of individual differences in the *KE*, which has been described in previous studies^37^. In order to properly model these individual differences, we have drawn conclusions from hierarchical, mixed-modeling for both behavioral and imaging data.

Further, the decrease in intentionality ratings for negative conditions over time and the increase in intentionality ratings for positive conditions is replicated from Experiment 1, and overall is consistent with a two-mechanism model, where practice effects push ratings for negative and positive consequences in opposite directions towards an implicit baseline. A one-mechanism model would alternatively predict that ratings for both negative and positive consequences would move in the same direction—either both increase or decrease.

Alternatively, one could interpret such practice effects as arising from demand effects. In fact, one major theory within the literature, the *conversational pragmatics theory*, holds something very similar to this. Participants provide an answer that is most consistent with their inference about the questioner’s desired information. However, reducing the mechanism of the *KE* to solely demand effects does not sufficiently explain why such demand effects would diminish over time. More importantly, demand effects do not explain our converging behavioral and neural evidence demonstrating two distinct processes for the asymmetry. In the case of negative consequences, for instance, the practice effects would more likely be due to emotional desensitization rather than diminishing demand effects over time. Further, demand effects would not explain the role we have found moral judgments to play in mediating the two distinct processes of the asymmetry.

Previous studies on the *KE* have demonstrated asymmetric judgments on a variety of entities including desire^12^, knowledge^38^, causation^39^, in addition to intentionality^10^. For instance, Gugliemo and Malle have shown that ascriptions of desire are higher for actions leading to negative consequences than positive consequences^12^. We speculate that one or more of these asymmetries may actually mediate the asymmetry observed for intentionality, where moral judgments potentially have an influence on ascriptions of desire, which could then have consequences on ascriptions of intentionality. Although future studies may extend our data to these other judgments, we remain focused on the implications that the *KE* has on intentionality, especially considering that the asymmetry in intentionality is the most validated and thoroughly characterized effect in the literature at this point^10^. Further, its implications on moral and legal theory hold regardless of whether it is a proximal effect or whether it is a downstream effect of one of these other entities.

For negative consequences, our data are not only consistent with theories describing the *KE* as an emotionally-based mechanism of motivational bias^15^,^40^,^41^, but also address a major criticism heretofore: the lack of any positive evidence for these theories^10^. A second criticism is also addressed, coming from a previous study that showed an intact *KE* in a population of patients with blunted affect associated with ventromedial prefrontal cortex (VMPFC) lesions^42^. We demonstrate that the crucial emotional signals that lead to ascriptions of intentionality may be generated in the amygdala (which were intact in the patients of the previous study) rather than the VMPFC.

The conclusions regarding positive consequences and its underlying mechanisms provide a new focus for future research. Just as for negative conditions, intentionality judgments for positive conditions are influenced by a motivational bias. However, instead of motivation to ascribe blame and intentionality, participants are motivated to withhold credit, and consequently, withhold intentionality, because of a generally negative attitude towards the agent (an attitude shared across both conditions). This attitude arises from the fact that the agent expresses an indifferent attitude towards something good, like helping the environment. Supplemental survey data from online testing supports this: 76% of participants had some sort of negative attitude towards the CEO in the positive consequence scenario (see *Methods*). However, the motivation to withhold credit and intentionality is not driven by emotion. Instead, statistical norms play the major role. Statistical norms have been implicated in previous work on the use of causal language^39^, and future work may elucidate whether judgments of causality serve as the critical mediator in the denial of intentionality.

More broadly, we demonstrate a mechanism by which moral judgments can paradoxically influence judgments of intentionality. Previous work has integrated behavioral and neural data to study the mechanisms of moral judgment^43^,^44^,^45^,^46^, and more specifically, the influence of intentions on moral judgment^4^,^29^,^47^,^48^,^49^,^50^. We extend this literature by developing a novel conceptualization for intentionality judgment and its neural mechanisms, and we utilize this framework to identify how the commonly conceived directionality between intentions and moral judgments can be reversed.

A revised model of intentionality judgment, arising from this and previous interactions between philosophy and empirical studies, can have direct implications for the legal system where questions of intentionality remain foundational. For instance, criminal law implicitly assumes that judges and jurors make independent judgments about a defendant’s *actus reus* (“guilty act”) and *mens rea* (“guilty mind”). However, it seems that judgments of one may influence the other: the consideration of a particularly egregious act (e.g., killing) may bias judges and jurors towards ascribing a certain associated mental state (e.g., intentional killing)^15^.

Beyond the legal system, our findings also have important implications for a central principle in moral theory and practice, the doctrine of double effect (DDE). The doctrine asserts that it is morally wrong to cause harm intentionally in circumstances where it would not be morally wrong to cause harm unintentionally^51^. The DDE thus places intentionality ascriptions at the very foundation of moral reasoning. This principle was suggested by St. Augustine^52^ and St. Thomas Aquinas^3^ in the Middle Ages and since then has remained central to Catholic moral teachings as well as to many secular theories in moral philosophy^53^ and moral psychology^4^. In recent years, the DDE has been cited in arguments against terror bombing^54^, against nuclear retaliation on cities during the Cold War^55^, against some forms of contraception and abortion^56^, and against active euthanasia and assisted suicide^57^—all on the grounds that these practices involve causing death intentionally. However, if ascriptions of intentionality already presuppose a prior moral judgment about the value of consequences, as our data demonstrate, then the DDE would be threatened with circularity, showing that it cannot be fundamental in moral theory. The moral mechanisms of the *KE* could thus force reconsideration of core tenets of moral theory in theology and philosophy.

## Methods

### Experiment 1

Across four different rounds of experimentation (*N* = 71, 74, 68, 70; mean age: 23, 64% female), 283 participants were recruited from Duke University and the surrounding community. All participants provided informed consent as part of a protocol approved by the Institutional Review Board of Duke University. Additionally, all methods were carried out in accordance with the approved protocol.

Participants completed self-paced and more verbose versions of a subset of 30 vignettes (15 pairs) drawn from the comprehensive pool of vignettes presented in the Supplementary Methods. Participants answered on a scale from 1 (Not Intentionally at All) to 8 (Completely Intentionally). The entire vignette and question were presented within one screen. The end-labels of this scale were counterbalanced across trials. Word count across negative and positive scenarios was balanced. For each successive round of experimentation, we sought to identify whether various individual-difference measures correlated with intentionality judgments. This included attempted replications of previously reported associated measures^21^,^23^, as well as others drawn from several fields including personality psychology, decision making, and moral psychology (Supplementary Methods).

No participants were excluded from analysis. We initially ran a paired *t*-test to assess for replication of the asymmetry of the *KE* (excluding pairs with missing data). We then fit a hierarchical, mixed-effects model^58^ in order to account for the repeated-measures nature of our design (i.e. the nesting of vignette trials within participants). These models are robust to randomly missing trial data, permit non-normally distributed outcomes (i.e. log-normally distributed response times), and allowed for the simultaneous examination of trial-varying and participant-varying effects. We fit models using SAS 9.3 Proc GLIMMIX^59^ with adaptive Gaussian quadrature estimation^60^. The residual degrees of freedom were divided into between-participant and within-participant portions^61^. Continuous independent variables were mean-centered, and random intercepts were included reflecting individual-differences in participant means. Across Experiments 1 and 3, random slopes provided little additional explanatory power and were excluded for parsimony. In each case, we analyzed null models (random intercept, no trial-level regressors) to estimate the intraclass correlation (participant-level variance/participant + trial level variance), reflecting the proportion of total variance accounted for by the clustering of responses by participant. For all models, these values were substantively and statistically large (0.22 and 0.20 for Exp. 1 and 3, respectively), justifying a mixed-model repeated-measures approach, as our measurements violated the independence assumption. Separate models were fit for the dependent variables, intentionality rating (Supplementary Equations 1, 3, and 4) and decision time (Supplementary Equations 2, 3, and 5). Decision time residuals appeared log-normally distributed, so we fit a generalized linear mixed model using a log-normal distribution and an identity link function. Correlations between scale measures and mean participant vignette responses were also analyzed.

### Experiment 2

Through the online labor market, Amazon Mechanical Turk (AMT), 400 participants were recruited, redirected to Qualtrics, completed an online survey, and each paid $0.25 in total. In all, 386 participants correctly answered an open-ended, comprehension catch question and were included in subsequent analyses. Demographics data was not collected, since we found that an effective recruitment strategy was minimizing the length of task. Other studies have shown that the mTurk participants are 32.8 years old on average and 55% female^62^. All participants provided informed consent as part of an IRB exemption approved by the Institutional Review Board of Duke University.

All participants read and completed negative and positive versions of Scenario #4 (Supplementary Methods). This is based on the first and most commonly used *Knobe Effect* vignette in the literature^8^,^10^. Additionally, participants read a neutral condition vignette:

The chairman started a plan to increase revenue. He did not care at all about the effect the plan would have on the color of the product. He knew his plan would make the product yellow. Did the chairman intentionally change the color of the product?

The environment is not mentioned in this vignette because of its evocative nature; its omission allows for this condition to be low in salience (Supplementary Methods). Participants answered on a scale from 1 (Not at all intentional) to 8 (Completely Intentional). The entire vignette and question was presented within one screen. The end-labels of the scale were counterbalanced across scenarios and participants. Participants were also asked about the emotional salience of each of these vignettes: “How strongly did you emotionally react to the [environment/product color] being [harmed/helped/changed to yellow]? Participants answered on a scale from 0 (Not at all) to 10 (Extremely). The end-labels of the scale were also counterbalanced across scenarios and participants. Finally, participants were asked whether they had ever seen these scenarios before and about the color of the product, as a catch question, from the neutral condition.

Participants who did not correctly provide an answer to the catch question were excluded before data analysis. Ten participants reported that they had seen a scenario that was similar to the one presented for this study. Exclusion of these participants did not significantly change the results of our analysis, so the presented analyses include all participants. Planned paired *t*-tests were performed across all three pairs of conditions. Single regression analyses between salience and intentionality ratings for each valence condition were performed in JMP 10.

In a supplemental survey, 110 participants were given one of the versions of the same vignette stem as those described above (56 for negative and 54 for positive). The participants were also asked about their general attitude towards the agent of the vignette with the following answer choices: *Very Positive*, *Somewhat Positive*, *Neutral*, *Somewhat Negative*, and *Very Negative*.

### Experiment 3

According to the plan established before data analysis, twenty adults (mean age: 24, range: 18–32 years; 10 females) with normal or corrected-to-normal vision completed the study. We excluded four individuals from the final data analyses: one for an incidental anatomical finding, one for excessive head movement (>2 mm), and two for behavioral homogeneity precluding the inclusion of the parametric regressor in our GLM as described below. Prescreening excluded individuals with prior or current psychiatric or neurological illness. All participants provided written informed consent as part of a protocol approved by the Institutional Review Board of Duke University Medical Center.

Participants completed four 20-trial runs of our Knobe Effect task (Supplementary Methods). Forty unique scenarios (15 from Experiment 1) were constructed with a similar structure to those used in previous studies on the Knobe Effect, in which each scenario had two versions: negative and positive consequences (Supplementary Methods). To prevent participants from anticipating the moral valence of vignettes based upon task history, minor variations were incorporated into each of the scenarios across valence version, and the task was coded such that two versions of the same scenario did not appear in the same run in the fMRI scan. Vignettes were balanced for gender of agent and included both proper names of agents (e.g., “Bill”) and general titles (e.g., “the doctor”; Supplementary Methods). Participants were asked to answer on a scale from 1 to 8 with scale-end labels of “Not intentionally at all” and “Completely intentionally” randomized in left-right orientation trial-by-trial. There was a 2 second interval between trials. Different parts of the vignette were presented in isolation, as demonstrated in Fig. 1a. After the scanning session, participants were again shown the vignettes from scanning, but were prompted with three additional questions about *emotional reaction*, *statistical normativity*, and *moral judgment* (Supplementary Methods). A paired *t*-test and hierarchical mixed-effect models were fit in the same manner as in Experiment 1. Unlike the analysis for Experiment 1, decision times were not modeled and analyzed because the fMRI task restricted the pace by which participants could progress through and provide responses to the vignettes.

We tested various mediation models using trial-by-trial ratings for *emotional reaction*, *blame*, and *intentionality* for negative conditions and trial-by-trial ratings for *statistical normativity*, *credit*, and *intentionality* for positive conditions. The MBESS package for R^63^,^64^ was used to calculate 95% confidence intervals with non-parametric bootstrapping (10,000 samples). Hypothesis testing was performed at α = 0.05 by determining whether the bootstrapped 95% confidence interval was inclusive of 0 65. Further, we sought to test whether the “*emotional reaction*  → *blame* → *intentionality*” and “*statistical normativity* → *credit* → *intentionality*” mediation models had significantly higher indirect effects than “*emotional reaction* → *intentionality* → *blame*” and “*statistical normativity* → *intentionality* → *credit*,” respectively. To do this, we took the difference in randomized bootstrap samples from the two theories compared and similarly performed hypothesis testing at α = 0.05 by determining whether the 95% confidence interval of the difference in indirect effects was inclusive of 0. All mediations above were checked for interactions between the *X* (independent) variables and *M* (mediator) variables and possible moderator relationships were ruled out.

Functional MRI data were acquired using a 3T GE scanner with an 8-channel receiver using a spiral-in sensitivity encoding (SENSE) sequence. Four runs of 306 time points were acquired with TR = 1.58, TE = 30 ms, voxel size = 3.8 mm × 3.8 mm × 3.8 mm, field of view = 243 mm, and flip angle = 70°. FMRI analyses were conducted in a conventional manner using tools from FSL (FMRIB Software Library; Supplementary Methods).

Because of the repeated nature of our task, participants would have easily been able to predict the content of the subsequent “Question” epoch during the “Knowledge” epoch. Therefore, the design of our task made the assumption that most of the crucial neural processing leading to intentionality judgments would be made during the “Knowledge” period, and processing during the “Question” period would mostly be related to identifying the correct keypad response. For this reason, all presented analyses are taken from the “Knowledge” epoch when participants are first able to determine the moral valence of the vignette. However, an alternative model using a period starting at the onset of the “Knowledge” period and ending with the keypress (in the middle of the “Question” period) did not significantly change the nature of our results.

The first-level (within-run) analysis included two categorical regressors for moral valence of the consequence (positive vs. negative) and parametric regressors for normalized (within run and valence condition) participant ratings of intentionality. Participants providing consistent responses leading to rank deficient design matrices were excluded from the study (*N* = 2). An additional categorical regressor was included for the “Question” epoch corresponding to whether the participant used the right or left hand in providing a response. This was used both as a nuisance regressor and an internal check of the validity of analyses based upon appropriate laterality of motor cortex activation. Additional nuisance regressors were included for the “Action” and “Attitude” epochs. Second-level analyses (across-runs, within-participants) used a fixed-effects model, and third-level analyses (across-participants) used a mixed-effects model (FLAME 1). Reaction time was not used as a nuisance regressor for any of the analyses because the period of time for the Knowledge period of the task was fixed at 5 seconds. Since participants were able to predict the nature of the subsequent question by the onset of the Knowledge period after several trials, the RT would have had a floor set at 5 seconds.

Peak voxels were drawn from the Neurosynth reverse-inference map for the term “emotion.” Reverse inference maps indicate the specificity of relevant terms to specific brain coordinates and utilize a Bayesian statistic that controls for the number of studies associated with each term^66^. This yielded global peaks in the left (*z-*score = 17.4; MNI: (−20, −4, −16)) and right amygdala (*z*-score = 14.2; MNI: (22, −2, −14)). These peaks represent voxels most specifically associated with the term “emotion” throughout the entire brain^66^,^67^, and “emotion” is the most relevant reverse inference to be drawn from these coordinates. Other brain regions usually associated with emotion, including the ventromedial prefrontal cortex, insula, medial orbitofrontal cortex, and anterior cingulate cortex, all had markedly lower *z*-scores peaks (all *z-scores* < 7.0).

Further, the top Neurosynth terms associated with these two coordinates all had a direct relation to emotion processing (e.g., emotion, neutral, negative, fearful, etc.) with the exception of a cluster involving facial processing (i.e., faces, face, facial). However, the nature of our stimuli make it unlikely that such terms represent significant confounds in our reverse inference. In total, this suggests that of all these brain regions and possible psychological constructs, amygdala seems to be most specific for emotional processing. Spheres with 8 mm radii were drawn at these coordinates in left and right amygdala. These spheres served as ROIs for finding voxels that significantly correlated on a between-participants basis with intentionality ratings. After small volume correction within this ROI, the activity from significantly correlated voxels was used for independent mediation analyses described below.

We tested whether post-scan measures of *emotional reaction* to negative scenarios significantly mediated the relationship between activity in bilateral dorsal amygdala and intentionality rating at a between-subjects level. As negative controls, we also tested whether the same relationship held for positive consequences. The ROI analysis for positive consequences did not yield any significant voxels after small volume correction, so we obtained activation estimates from the same voxel coordinates that had been found in the negative consequence mediation analysis. We also performed a whole-brain analysis for voxels whose activities significantly correlated between-participants with mean intentionality ratings. This analysis yielded activation in left dorsolateral prefrontal cortex (L DLPFC) as well as several occipital regions. We used the significant cluster of activation from L DLPFC as another negative control for a mediation analysis. These neural mediation models were tested in a similar manner to those described above.

## Additional Information

**How to cite this article**: Ngo, L. *et al.* Two Distinct Moral Mechanisms for Ascribing and Denying Intentionality. *Sci. Rep.* **5**, 17390; doi: 10.1038/srep17390 (2015).

## Supplementary Material

Supplementary Information
