# Supplementary material for: Two Distinct Moral Mechanisms for Ascribing and Denying Intentionality
Source: Sci Rep. 2015 Dec 4;5:17390. doi: 10.1038/srep17390 (PMC4669441; doi:10.1038/srep17390)
Supplement: Supplementary Information [file srep17390-s1.doc]

Supplementary Information for

**Two Distinct Moral Mechanisms for Ascribing and Denying Intentionality**

Lawrence Ngo, Meagan Kelly, Christopher Coutlee, R. McKell Carter, Walter Sinnott-Armstrong, Scott A. Huettel*

*Correspondence to: [scott.huettel@duke.edu](mailto:xxxxx@xxxx.xxx)

**Experiment 1 Supplementary Methods.** Data collection occurred across four different rounds (*N*=71, *N*=74, *N*=68, and *N*=70). For the first round, participants subsequently completed scales for the Interpersonal Reactivity Index (IRI)1, Tendency to Forgive Scale (TTF)2, Machiavellianism Test (MACH-IV)3, Vengeance Scale4, and Affective Intensity Measure (AIM)5. In the second round, participants subsequently responded to the IRI, Profile of Mood States (POMS)6, Revised NEO Personality Inventory (NEO PI-R; only the extraversion subscale)7, PAL (Personal Altruism Level)8, and TTF. For the third round, participants completed the Cognitive Reflection Task (CRT)9, Rational-Experiential Inventory (REI-40)10, Moral Foundations Questionnaire (MFQ)11, AIM, POMS, IRI, TTF, NEO PI-R (extraversion), and BIS (Barratt Impulsiveness Scale)12. For the fourth round, participants completed the NEO-ex, MFQ, and CRT.

The model equations for our hierarchical, mixed-models are presented below, where *i* is trial, *j* is participant, *r* is the error, *DT* is decision time, *trial* is the number of trials the participant has previously seen, *γ*00 is the overall intercept, and *u*­0*j* is the random error component for deviation of the participant’s intercept from the overall intercept.

Level 1

[Supplementary Equation 1]:

$$Intentionality_{ij}=\beta_{0j}+\beta_{1}val_{ij}+\beta_{2}trial_{ij}+\beta_{3}val_{ij}trial_{ij}+r_{ij}$$

[Supplementary Equation 2]:

$$\ln(DT_{ij})=\beta_{0j}+\beta_{1}val_{ij}+\beta_{2}trial_{ij}+\beta_{3}val_{ij}trial_{ij}+r_{ij}$$

Level 2

For both dependent variables:

[Supplementary Equation 3]:

$$\beta_{0j}=\gamma_{00}+u_{0j}$$

Reduced form:

[Supplementary Equation 4]:

$$Intentionality_{ij}=\gamma_{00}+\beta_{0j}+\beta_{1}val_{ij}+\beta_{2}trial_{ij}+\beta_{3}val_{ij}trial_{ij}+u_{0j}+r_{ij}$$

[Supplementary Equation 5]:

$$\ln(DT_{ij})=\gamma_{00}+\beta_{0j}+\beta_{1}val_{ij}+\beta_{2}trial_{ij}+\beta_{3}val_{ij}trial_{ij}+u_{0j}+r_{ij}$$

**Experiment 2 Supplementary Methods.** Experiment 2 was conducted through an online data collection procedure through Amazon Mechanical Turk (AMT). AMT has become an increasingly popular experimental tool and provides access to a large sample pool shown to be considerably more diverse than a typical American college population. Though some weaknesses have been discussed13–15, numerous replication studies have demonstrated that data collected on AMT is highly reliable and consistent with other methods of data collection13–18.

Several elements of our task design further ensure the quality of our data. As described in more detail below, we only included variations on one vignette instead of many to ensure that the task burden was not high (designed to be 2-3 minutes in duration). We also only included participants who had a previous approval rate of greater than 98% on AMT. There is the concern that with the growing popularity of AMT, participants may have repeatedly encountered similar tasks from various research groups, including our own14. We included a question directly asking whether he or she had seen a survey similar in nature to ours before, and we employed mechanisms within AMT and Qualtrics to prevent the same user or IP address from completing the survey more than once. Finally, we limited our participants to only those from the United States. Though there has been growing concern that users outside the United States have been able to circumvent these restrictions15, the *KE* task has previously been shown to generalize well to other languages and cultures, particularly Hindi19.

An alternative low-salience, neutral vignette that was considered was “The CEO knew the plan would have no impact on the environment, though she did not care at all about the effect the plan would have on the environment.” However, we decided against using this vignette for several reasons. First, mention of the environment would have significant salience since it is a politically charged topic. This would have been counterproductive to the goal of having a low salience condition to test whether salience is the underlying mechanism. Second, the relevant question that participants would consider would be “Did the CEO intentionally not have an impact on the environment?” Previous work has elucidated the differences between positive acts and acts of omission 20,21, and this would have introduced a problematic confound.

**Experiment 3 Supplementary Methods.** For fMRI preprocessing and analysis using FSL, brain tissue was first isolated using the brain extraction tool22. The first six volumes of each analyzed run were discarded to account for magnetic stabilization. Differences in slice acquisition times were corrected using Fourier-space phase shifting. Spatial smoothing was performed with a Gaussian kernel with a full width at half maximum of 6 mm. Grand mean scaling was performed across datasets from each run of each participant. A high-pass temporal filter was applied with a Gaussian-weighted least-squares straight line fitting with =100 s. Functional images were registered to participants’ high-resolution structural images with FLIRT, and subsequently, to MNI standard space with FNIRT23. Head motion was corrected by realigning the time series to the middle volume using FLIRT23. All fMRI analyses were performed with FEAT (FMRI Expert Analysis Tool) Version 5.98, which is part of FSL (FMRIB’s Software Library). Time-series local autocorrelation correction was carried out with FILM24.

During a post-scanner session, participants responded to additional questions modeled after the following for each scenario. Results for question 1 were reverse coded to indicate the level of negative emotional reaction to the vignettes.

- How did the CEO’s harming (or helping) the environment make you feel? [-3=Very Negative to 3=Very positive]
- How much blame (or credit) does the CEO deserve for harming (helping) the environment? [1=No Blame at All to 8=Extreme Blame]
- About how many people out of 100 in the general population would have harmed (helped) the environment under these circumstances? [0 to 100]

The model equations for the hierarchical, mixed-models for Experiment 3 are presented below, where *i* is trial, *j* is participant, *r* is the error, *trial* is the number of trials the participant has previously seen, *emot* is the measure of *emotional reaction* (reverse-coded), *stat* is the measure of *statistical normativity*, γ00 is the overall intercept, and *u*­0*j* is the random error component for deviation of the participant’s intercept from the overall intercept.

Level 1

[Supplementary Equation 6]:

Level 2

[Supplementary Equation 7]:

Reduced form:

[Supplementary Equation 8]:


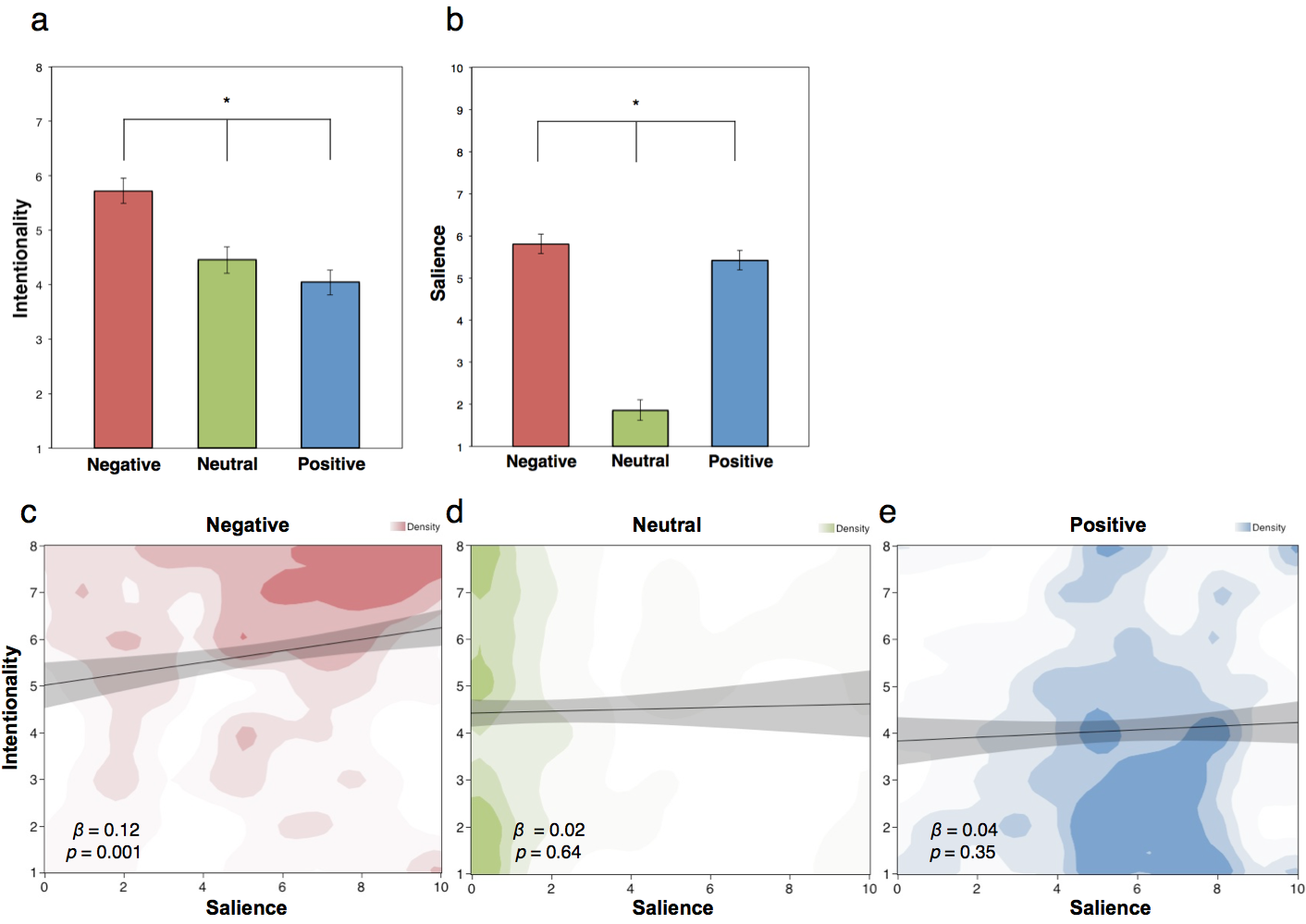


Supplementary Fig. 1. Emotional salience does not account for differences in intentionality ratings between outcomes with different emotional valence, but it does predict intentionality for negative outcomes. Participants (*N*=386) on AMT were presented three versions of scenario #4 differing in valence. (a) All pairwise comparisons among the three conditions were significant. Participants ascribed higher intentionality for negative compared to positive (paired *t*(385)=11.3, *p* < 0.0001), higher for negative compared to neutral (paired *t*(385)=8.19, *p* <0.0001) and lower intentionality to positive compared to neutral (paired *t*(385)=2.58, *p*<0.01). The data from negative and positive conditions were also presented in Fig. 1B. (b) The neutral condition had significantly lower ratings of salience than negative (paired *t*(385)=18.03, *p* <0.0001) and positive conditions (paired *t*(385)=-17.8, *p* <0.0001). Negative conditions did have higher salience ratings than those for positive conditions (paired *t*(385)=2.28, *p*=0.02). Error bars indicate 95% confidence interval. (c) For negative conditions, salience ratings were positively correlated with those for intentionality. The same was not found in neutral conditions (d) or in positive conditions (e). Density plots are overlaid with a regression line with 95% confidence interval. *All pairwise comparisons are significantly different from one another according to a paired t-test.


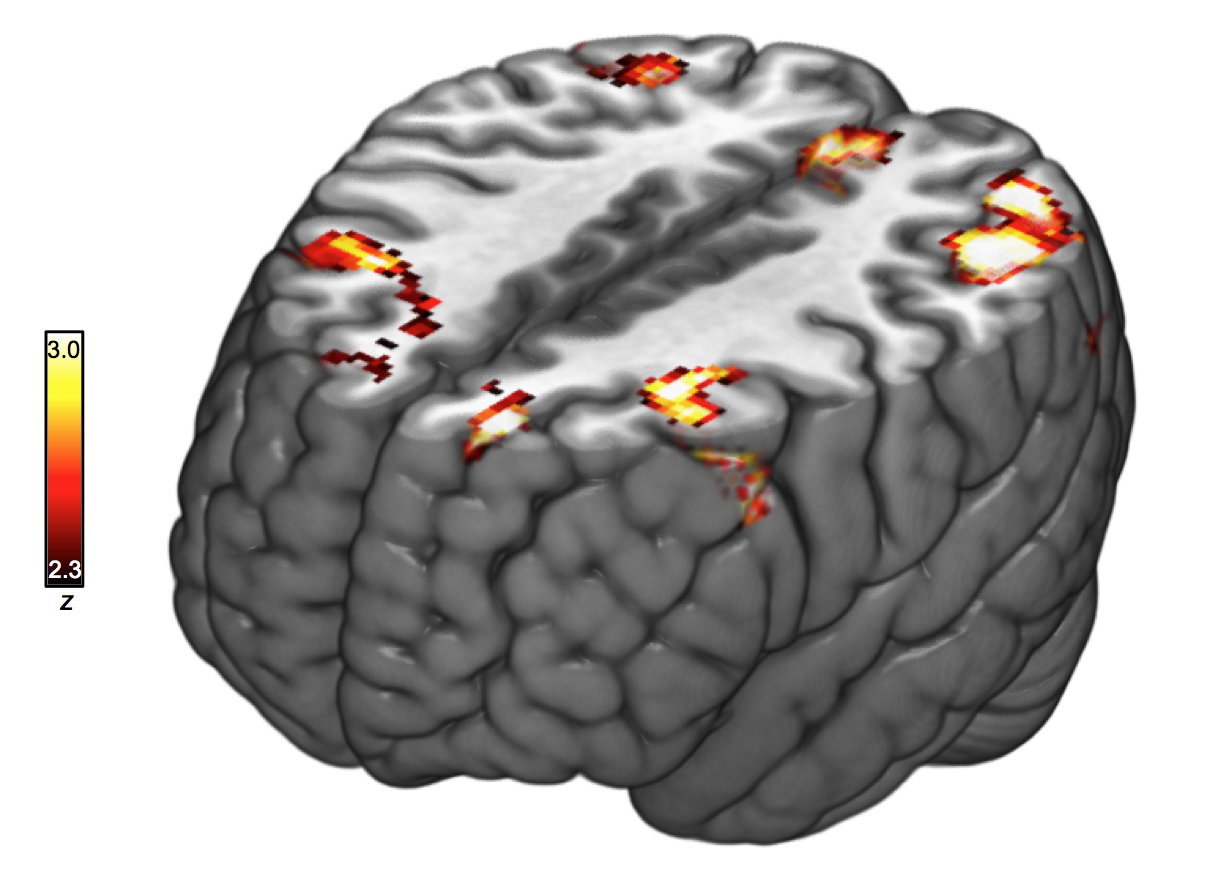


Supplementary Fig. 2. Direct contrast of positive > negative consequences. Regions including bilateral middle frontal gyrus, bilateral temporoparietal junction, and precuneus were more activated for positive consequences than for negative consequences during the “knowledge” epoch with a cluster threshold of *z* > 2.3 and a whole-brain cluster correction of *p* < 0.05. No regions were significantly activated for negative > positive consequences. Activations are presented in Supplementary Table 5.


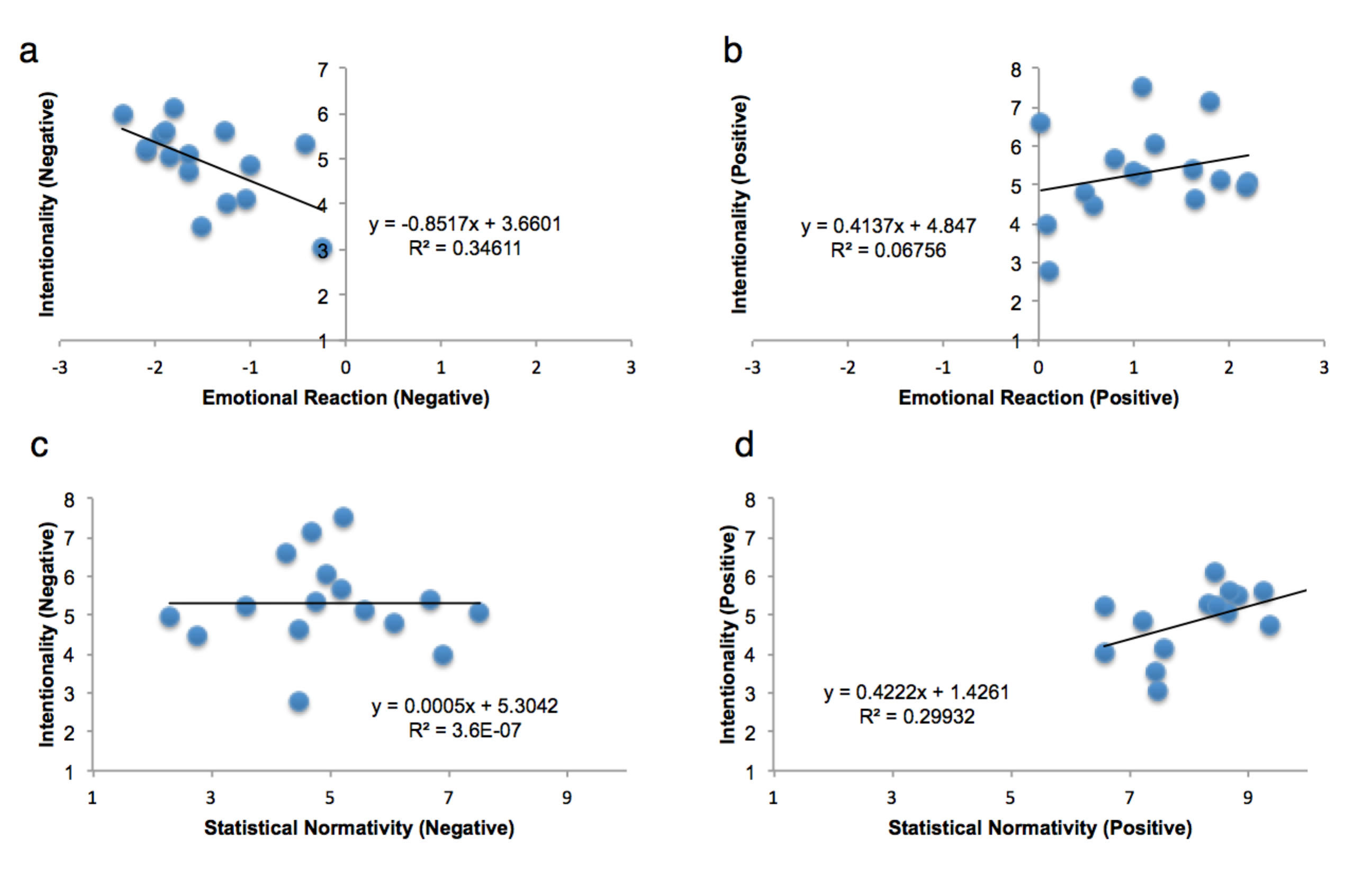


Supplementary Fig 3. Unmodeled participant level means from Experiment 3. Care must be taken in interpreting these plots since they do not control for participant-level variance and participant + trial level variance, which are taken into account in the hierarchical model employed in the paper.However, survey of the data reveals that no significant ceiling effects are contributing to the double dissociation that is described in our full models. (a) Emotional Reaction and Intentionality for Negative Conditions. (b) Emotional Reaction for Positive Conditions. (c) Statistical Normativity and Intentionality for Negative Conditions. (d) Statistical Normativity and Intentionality for Positive Conditions.

*Scatterplots have been added to the supplemental material and are not remarkable for any significant floor effects for either of the two conditions that did not show significant effects in our hierarchical model: emotional reaction in positive conditions and statistical normativity in the negative condition. However, care must be taken in interpreting these plots since they do not* *control for participant-level variance and participant + trial level variance, which are taken into account in the hierarchical model employed in the paper.*

Supplementary Table 1. Results from the hierarchical, mixed-effects model of ratings of intentionality from experiment 1 (self-paced, long-form, Campus Behavioral experiment).

| **Fixed Effects** | **Coefficient** | **SE** | **DF** | ***t*** | ***p*** |
| --- | --- | --- | --- | --- | --- |
| Intercept (Mean Centered) | 3.57 | 0.07 | 282 | 53.80 | **<0.0001** |
| Scen. Valence (Neg. Vs. Pos.) | 1.41 | 0.04 | 282 | 36.44 | **<0.0001** |
| Scen. Order × Scenario Valence Interaction (Neg. vs. Pos.) | -0.01 | 0.004 | 8049 | -3.15 | **0.005** |
| Scen. Order (Slope for Neg. Scen.) | -0.006 | 0.003 | 8049 | -2.05 | **0.04** |
| Scen. Order (Slope for Pos. Scen.) | 0.007 | 0.003 | 8049 | 2.44 | **0.01** |
|  |  |  |  |  |  |
|  |  |  |  |  |  |
| **Random Effects** | **Coefficient** | **SE** |  |  |  |
| Participant-level variance | 1.03 | 0.010 |  |  |  |
| Trial-level variance | 3.13 | 0.05 |  |  |  |

There was a main effect of valence such that negative conditions had higher ratings than those for positive conditions. Further, the increased power of this experiment compared to the fMRI experiment allowed for evidence of a full interaction between scenario order and valence, such that negative conditions became less intentional over time while positive conditions became more positive. One binary regressor was included to model Scenario Valence, but the table summarizes results from two analyses with either positive or negative scenarios set as the categorical reference group to allow for the examination of continuous effects in each group. The intraclass correlation calculated from the null model was 0.22, strongly supporting our use of mixed models.

Supplementary Table 2. Results from the hierarchical, mixed-effects model of ratings of decision time from experiment 1 (self-paced, long-form, Campus Behavioral experiment).

| **Fixed Effects** | **Coefficient** | **SE** | **DF** | ***t*** | ***p*** |
| --- | --- | --- | --- | --- | --- |
| Intercept (Mean Centered) | 2.99 | 0.02 | 282 | 125.66 | **<0.0001** |
| Scen. Valence (Neg. Vs. Pos.) | -0.07 | 0.01 | 282 | -7.70 | **<0.0001** |
|  |  |  |  |  |  |
| Scen. Order × Scen. Valence Interaction (Neg. vs. Pos.) | 0.001 | 0.001 | 8056 | 0.52 | **0.60** |
| Scen. Order (Slope for Neg. Scen.) | -0.02 | 0.001 | 8056 | -27.81 | **<0.0001** |
| Scen. Order (Slope for Pos. Scen.) | -0.02 | 0.003 | 8056 | -28.55 | **<0.0001** |
|  |  |  |  |  |  |
| **Random Effects** | **Coefficient** | **SE** |  |  |  |
| Participant-level variance | 0.15 | 0.013 |  |  |  |
| Trial-level variance | 0.19 | 0.003 |  |  |  |

Participants took significantly longer to respond to positive conditions than negative conditions. Though participants exhibited practice effects with decreasing decision times over successive trial, there was no interaction of this practice effect with valence of the condition. One binary regressor was included to model Scenario Valence, but the table summarizes results from two analyses with either positive or negative scenarios set as the categorical reference group to allow for the examination of continuous effects in each group. The intraclass correlation calculated from the null model (e.g. the proportion of variance attributable to participant effects) was 0.38, strongly supporting our use of mixed models. As this model reflects the results of a generalized linear mixed model using a lognormal distribution and identity link function, the coefficient estimates should not be directly interpreted (the *t* and *p* values are interpretable as usual). Interpretable estimate values were obtained by calculating predicted effects for groups by adding/subtracting estimate values, then computing the inverse natural log of the calculated value to express the predicted values in seconds, the original unit scale. Finally we took differences of these interpretable values between conditions. Using this method, the intercept for help scenarios was 19.89 seconds, decision times for harm scenarios were on average 1.49 seconds faster, and each additional scenario was associated with a 0.58 second decrease in decision time.

Supplementary Table 3. Results from the hierarchical, mixed-effects model of ratings of intentionality from experiment 3.

| **Fixed Effects** | **Coefficient** | **SE** | **DF** | ***t*** | ***p*** |
| --- | --- | --- | --- | --- | --- |
| Intercept (Mean Centered) | 4.52 | 0.22 | 15 | 20.27 | **<0.0001** |
| Scen. Valence (Neg. vs. Pos.) | 0.12 | 0.15 | 15 | 0.82 | **0.43** |
| *Emotion* × Scen. Valence Interaction (Neg. vs. Pos.) | 0.45 | 0.11 | 1209 | 4.32 | **<0.0001** |
| *Emotion* (Slope for Neg. Scen.) | 0.44 | 0.07 | 1209 | 6.30 | **<0.0001** |
| *Emotion* (Slope for Pos. Scen.) | -0.01 | 0.07 | 1209 | -0.16 | 0.87 |
|  |  |  |  |  |  |
| *Stat.* *Norm.*× Scen. Valence Interaction (Neg. vs. Pos.) | -0.23 | 0.05 | 1209 | -4.59 | **<0.0001** |
| *Stat. Norm.* (Slope for Neg. Scen.) | -0.03 | 0.03 | 1209 | -0.80 | 0.42 |
| *Stat. Norm.* (Slope for Pos. Scen.) | 0.20 | 0.04 | 1209 | 5.39 | **<0.0001** |
|  |  |  |  |  |  |
| Scen. Order × Scen. Valence Interaction (Neg. vs. Pos.) | -0.01 | 0.003 | 1209 | -2.34 | **0.02** |
| Scen. Order (Slope for Neg. Scen.) | -0.004 | 0.003 | 1209 | -1.27 | 0.20 |
| Scen. Order (Slope for Pos. Scen.) | 0.01 | 0.003 | 1209 | 2.05 | **0.04** |
|  |  |  |  |  |  |
| Reversed Rating Scale | 0.11 | 0.10 | 15 | 1.16 | 0.27 |
|  |  |  |  |  |  |
| **Random Effects** | **Coefficient** | **SE** |  |  |  |
| Participant-level variance | 0.58 | 0.22 |  |  |  |
| Trial-level variance | 2.91 | 0.12 |  |  |  |

Significant *valence* × *emotional reaction* and *valence* × *statistical normativity* interactions showed a double dissociation in mechanism of *Ascription* and *Denial* as illustrated in Fig. 2A. There was no main effect of scenario valence after accounting for *emotional reaction* and *statistical normativity*. Further, a significant scenario order × *valence* interaction showed that ratings for positive conditions significantly increased over time while there was a trend for decreasing ratings for negative conditions. One binary regressor was included to model Scenario Valence, but the table summarizes results from two analyses with either positive or negative scenarios set as the categorical reference group to allow for the examination of continuous effects in each group. The *emotional reaction* metric was reverse coded. The intraclass correlation calculated from the null model was 0.20, strongly supporting our use of mixed models.

Supplementary Table 4. Mediation models of the neural mechanisms for the ascription of intentionality for negative consequences.

|  | Indirect Effect (Δ *β*) Estimate | 95% Confidence [Lower, Upper] |
| --- | --- | --- |
| Amygdala  *Emotion* *Intentionality*  Negative Consequences  Positive Consequences | 0.80*  0.07 | [0.07, 2.02]  [-0.20, 0.65] |
| L DLPFC  *Emotion*  *Intentionality*  Negative Consequences | 0.57 | [-0.06, 1.45] |

For negative consequences, the association between amygdala activation and intentionality ratings was mediated by emotional reactions. As a negative control, we found that the same relationship did not hold for positive consequences. Also as a negative control, we explored whether emotion mediated the relationship that was found between the left dorsolateral prefrontal cortex (L DLPFC) and intentionality ratings (Supplementary Table 7). Consistent with stronger predictions for an association for emotional reaction with the amygdala compared to the L DLPFC, we did not find emotion to mediate this relationship.

**Supplementary Table 5. Activations for main contrast for the “knowledge” regressor for positive outcomes > negative outcomes passing whole-brain cluster-correction at *p* < 0.05 (cluster threshold of *z*** > 2.3).

| Region | x | y | z | Voxels | Z-max |
| --- | --- | --- | --- | --- | --- |
| L Temporoparietal Junction | -44 | -54 | 38 | 1345 | 3.67 |
| L Middle Frontal Gyrus | -42 | 20 | 36 | 1238 | 3.61 |
| R Middle Frontal Gyrus | 40 | 16 | 48 | 842 | 3.43 |
| R Temporoparietal Junction | 46 | -58 | 36 | 458 | 3.68 |
| Precuneus | -4 | -68 | 40 | 456 | 3.52 |

No areas of significant activation were found for negative outcomes > positive outcomes. Coordinates are mm in MNI space. The SPM is presented in Supplementary Fig. 2.

**Supplementary Table 6. Moral judgments of blame and credit serve as inputs for intentionality ascription in both *Ascription* and *Denial*** (models 1 and 4).

|  | Estimates | 95% Confidence [Lower, Upper] |
| --- | --- | --- |
| 1. *Emotion*  *Blame* *Intentionality*  2. *Emotion*  *Intentionality*  *Blame*  3. Difference in 1 and 2 | Δ *β* = 0.30  Δ *β* = 0.09  Δ (Δ *β*) = 0.21 | [0.18, 0.43]  [0.05, 0.13]  [0.08, 0.34] |
| 4. *Stat. Norm.*  *Credit* *Intentionality*  5. *Stat*. *Norm*.  *Intentionality*  *Credit*  6. Difference in 1 and 2 | Δ *β* = 0.10  Δ *β* = 0.04  Δ (Δ *β*) = 0.06 | [0.05, 0.14]  [0.02, 0.06]  [0.01, 0.10] |

Models 2 and 5 are consistent with intentionality serving as an input for blame as has been previously studied. However, our data suggest that the directionality of the former class of models (1 and 4) have significantly higher mediation effects than the latter (2 and 5), since the difference in randomized bootstrap samples from models 1 and 2 and from models 4 and 5 both yield confidence intervals exclusive of 0.

**Supplementary Table 7.** Activations for main effect of negative outcomes for the “knowledge” regressor that were correlated with mean intentionality ratings for negative outcomes.

| Region | x | y | z | Voxels | Z-max |
| --- | --- | --- | --- | --- | --- |
| **L Occipital Fusiform Gyrus** | **-20** | **-68** | **-14** | **192** | **4.53** |
| **L DLPFC** | **-42** | **32** | **26** | **191** | **4.58** |
| **R Cerebellum** | **42** | **-62** | **-28** | **127** | **4.04** |
| R Frontal Pole | 42 | 52 | 12 | 113 | 4.29 |
| R Amygdala | 18 | -2 | -10 | 24 | 3.91 |
| R Occipital Fusiform Gyrus | 16 | -84 | -20 | 22 | 4.13 |
| Lingual Gyrus | 4 | -88 | -16 | 12 | 3.43 |
| L Supplementary Motor Cortex | -8 | 8 | 56 | 9 | 3.76 |
| R Cerebellum | 36 | -52 | -40 | 9 | 3.29 |
| L Amygdala | -18 | 0 | -12 | 8 | 3.72 |
| L Cerebellum | -16 | -78 | -30 | 7 | 3.55 |
| R Lateral Occipital Cortex | 36 | -66 | 60 | 6 | 3.63 |
| L Cerebellum | -4 | -80 | -38 | 6 | 3.3 |

For illustrative purposes, all indicated regions passed a voxel significance threshold of *p* < 0.001 uncorrected with a contiguous cluster extent greater than 5 voxels. Coordinates are mm in MNI space. Bolded clusters passed whole-brain correction at *p* < 0.05 (cluster threshold *z* > 2.3).

Experimental Stimuli: Vignettes

1.

Bill released a gadget to make a deadline.

Bill did not care at all about the effect the gadget would have on babies.

Bill knew his gadget would kill babies.

Did Bill intentionally cause the death of babies?

2.

Robyn released an invention to make a deadline.

Robyn did not care at all about the effect the invention would have on toddlers.

Robyn knew her invention would help toddlers.

Did Robyn intentionally help toddlers?

3.

The CEO started a plan to increase profits.

She did not care at all about the effect the plan would have on the environment.

She knew her plan would harm the environment.

Did the CEO intentionally harm the environment?

4.

The chairman started a plan to increase revenue.

He did not care at all about the effect the plan would have on the environment.

He knew his plan would help the environment.

Did the chairman intentionally help the environment?

5.

Roger enacted a financial scheme to buy a house.

Roger did not care at all about the effect the scheme would have on old retirees.

Roger knew his plan would bankrupt old retirees.

Did Roger intentionally bankrupt old retirees?

6.

Renee enacted a financial scheme to buy a car.

Renee did not care at all about the effect the scheme would have on old retirees.

Renee knew her plan would help old retirees.

Did Renee intentionally help old retirees?

7.

Natasha renovated the country club to improve its appearance.

Natasha did not care at all about the effect this would have on membership costs.

Natasha knew her renovation would increase membership costs.

Did Natasha intentionally increase membership costs?

8.

Floyd renovated the golf course to improve its playability.

Floyd did not care at all about the effect this would have on customer satisfaction.

Floyd knew his renovation would increase customer satisfaction.

Did Floyd intentionally increase customer satisfaction?

9.

The mayor diverted water to Oldtown to gain votes.

He did not care at all about the effect this would have on Newtown.

He knew diverting the water would deprive Newtown of water.

Did the mayor intentionally deprive Newtown of water?

10.

The councilwoman diverted water to her town to win an election.

She did not care at all about the effect this would have on food production.

She knew diverting the water for his town would increase food production.

Did the councilwoman intentionally increase food production?

11.

The CEO enacted a plan to increase profits.

She did not care at all about the effect the plan would have on flooding.

She knew her plan would increase flooding.

Did the CEO intentionally increase flooding?

12.

The company owner enacted a plan to increase profits.

He did not care at all about the effect the plan would have on flooding.

He knew his plan would reduce flooding.

Did the CEO intentionally reduce flooding?

13.

The airplane bomber bombed a factory to reduce enemy’s steel production.

He did not care at all about the effect the bombing would have on innocent civilians.

He knew his bombing would kill innocent civilians.

Did the airplane bomber intentionally kill innocent civilians?

14.

The bomber pilot bombed a facility to reduce the enemy’s iron production.

She did not care at all about the effect the bombing would have on the townsfolk.

She knew her bombing would liberate the townsfolk.

Did the bomber pilot intentionally liberate the townsfolk?

15.

The university president enacted a plan to increase business school funding.

He did not care at all about the effect the plan would have on the medical school.

He knew his plan would cut funding for the medical school.

Did the university president intentionally cut funding for the medical school?

16.

The athletic director enacted a plan to increase basketball funding.

She did not care at all about the effect the plan would have on the soccer team.

She knew her plan would boost funding for the soccer team.

Did the athletic director intentionally boost funding for the soccer team?

17.

Jenny spread weed killer to protect her crops.

Jenny did not care at all about the effect this would have on Susie-Ann’s crops.

Jenny knew her pesticide would harm Susie-Ann's crops.

Did Jenny intentionally harm her neighbor's crops?

18.

Stanley spread anti-fungals to protect his crops.

Stanley did not care at all about the effect this would have on Billy-Bob’s crops.

Stanley knew his anti-fungals would protect Billy-Bob’s crops.

Did Stanley intentionally protect Billy-Bob’s crops?

19.

Kate placed her uncle in a nursing home to avoid being his caretaker.

Kate did not care at all about the effect the placement would have on her uncle.

Kate knew placing him in a nursing home would make him extremely unhappy.

Did Kate intentionally make her uncle unhappy?

20.

Jared placed his aunt in a nursing home to avoid being her caretaker.

Jared did not care at all about the effect the placement would have on his aunt.

Jared knew placing his aunt in a nursing home would make her extremely happy.

Did Jared intentionally make his aunt happy?

21.

Tim installed a light display to decorate his house.

Tim did not care at all about the effect the display would have on the neighbors.

Tim knew his light display would hamper the neighbors' stargazing.

Did Tim intentionally hamper the neighbors' stargazing?

22.

Tricia installed a lighting array to decorate her yard.

Tricia did not care at all about the effect the array would have on neighborhood kids.

Tricia knew her light display would help the kids to play ball at night.

Did Tricia intentionally help the kids play ball at night?

23.

The scientist released a drug to gain profit.

She did not care at all about the effect the drug would have rates of cancer.

She knew her drug would increase the rate of cancer.

Did the scientist intentionally increase the rate of cancer?

24.

The scientist released a drug to make a deadline.

He did not care at all about the effect the drug would have on rates of heart attacks.

He knew his drug would decrease the rate of heart attacks.

Did the scientist intentionally decrease the rate of heart attacks?

25.

Joe opened a kiosk to make more money.

Joe did not care at all about the effect the kiosk would have on nearby vendors.

Joe knew his kiosk would harm nearby vendors.

Did Joe intentionally harm nearby vendors?

26.

Helen opened a new store to increase revenue.

Helen did not care at all about the effect the store would have on other businesses.

Helen knew her store would help other businesses.

Did Helen intentionally help other businesses?

27.

Marta vacuumed to clean her floor.

Marta did not care at all about the effect the vacuum would have on her dog.

Marta knew her vacuuming would distress her dog.

Did Marta intentionally distress her dog?

28.

Phil waxed his floor to make it shiny.

Phil did not care at all about the effect the waxing would have on his cat.

Phil knew his waxing would entertain his cat.

Did Phil intentionally entertain his cat?

29.

Jacob threw a party to be more popular.

Jacob did not care at all about the effect this would have on his roommate, Curtis.

Jacob knew his party would make Curtis fail the morning's exam.

Did Jacob intentionally make Curtis fail the morning's exam?

30.

Rachel threw a party to have fun.

Rachel did not care at all about the effect this would have on her roommate, Jackie.

Rachel knew her party would help Jackie make new friends.

Did Rachel intentionally help Jackie make new friends?

31.

Russell planted a tree to decorate his yard.

Russell did not care at all about the effect the tree would have on his neighbor.

Russell knew his tree would make his neighbor unhappy.

Did Russell intentionally make his neighbor unhappy?

32.

Vicky planted a tree to have fruit in the fall.

Vicky did not care at all about the effect the tree would have on her neighbor.

Vicky knew her tree would make her neighbor happy.

Did Vicky intentionally make her neighbor happy?

33.

Brenda cut spending at the animal shelter to increase her salary.

Brenda did not care at all about the effect the cut would have on the shelter.

Brenda knew cutting spending would cause a dog at the shelter to be put down.

Did Brenda intentionally cause the dog to be put down?

34.

Billy cut spending at the homeless shelter to increase his pay.

Billy did not care at all about the effect the cut would have on the shelter.

Billy knew cutting spending would help the shelter run more efficiently.

Did Billy intentionally help the shelter run more efficiently?

35.

The Surgeon General implemented the policy to keep his position.

He did not care at all about the effect the policy would have on rates of influenza.

He knew his policy would increase rates of influenza.

Did the Surgeon General intentionally increase rates of influenza?

36.

The Defense Secretary implemented the policy to remain politically popular.

She did not care at all about the effect the policy would have on rates of deaths.

She knew her policy would decrease deaths.

Did the Defense Secretary intentionally decrease deaths?

37.

Carolyn enacted the plan to increase earnings.

Carolyn did not care at all about the effect the plan would have on her employees.

Carolyn knew her plan would make employees unhappy.

Did Carolyn intentionally make employees unhappy?

38.

Christopher enacted the plan to increase earnings.

Christopher did not care at all about the effect the plan would have on his employees.

Christopher knew his plan would make employees happy.

Did Christopher intentionally make employees happy?

39.

Tina built a road to increase traffic to her country store.

Tina did not care at all about the effect this would have on a nearby 1000-year-old tree.

Tina knew her new road would cause the 1000-year-old tree to die.

Did Tina intentionally cause the 1000-year-old tree to die?

40.

Ronald bought a trolley to increase visitors to his theme park.

Ronald did not care at all about the effect this would have on a nearby monument.

Ronald knew his new road would make the monument famous.

Did Ronald intentionally make the monument famous?

41.

Jerry hunted animals to earn a living.

Jerry did not care at all about the effect this would have on endangered reindeer.

Jerry knew his hunting would cause the reindeer to become extinct.

Did Jerry intentionally cause the reindeer to become extinct?

42.

Linda trapped animals to do research.

Linda did not care at all about the effect this would have on endangered monkeys.

Linda knew her trapping would save the monkeys from their predators.

Did Linda intentionally save the monkeys from their predators?

43.

Irene decided to induce labor to reduce her pregnancy pains.

Irene did not care at all about the effect this would have on her baby's health.

Irene knew inducing labor would be bad for her baby's health.

Did Irene intentionally worsen her baby's health?

44.

Holly decided to induce labor to get her pregnancy over with.

Holly did not care at all about the effect this would have on her baby's health.

Holly knew inducing labor would improve her baby's health.

Did Holly intentionally improve the health for her baby?

45.

Eugene screams during a tennis match to express his excitement.

Eugene did not care at all about the effect this would have on the tennis player.

Eugene knew his yelling would cause the tennis player to lose.

Did Eugene intentionally cause the tennis player to lose?

46.

Margaret yells out during a golf tournament to express her excitement.

Margaret did not care at all about the effect the yelling would have on the golfer.

Margaret knew her yelling would cause golfer to win.

Did Margaret intentionally cause the golfer to win?

47.

Rebecca protests in support of political prisoners to get on TV.

Rebecca did not care at all about the effect her protests would have on the prisoners.

Rebecca knew her protests would cause the execution of the political prisoners.

Did Rebecca intentionally cause the execution of the political prisoners?

48.

Sean protests in support of the death row inmate to get onto newspapers.

Sean did not care at all about the effect this would have on the death row inmate.

Sean knew his protests would cause the release of the death row inmate.

Did Sean intentionally cause the release of the death row inmate?

49.

Curtis released the documents to gain publicity.

Curtis did not care at all about the effect this would have on his friend's reputation.

Curtis knew the documents would ruin his friend's reputation.

Did Curtis intentionally ruin his friend's reputation?

50.

Lori released the photos to gain news coverage.

Lori did not care at all about the effect this would have on her boss’s reputation.

Lori knew the photos would redeem her boss’s reputation.

Did Lori intentionally redeem her boss’s reputation?

51.

Clara enacted the new lunch plan to cut costs.

Clara did not care at all about the effect this would have on the health of school children.

Clara knew her new lunch plan would harm the health of school children.

Did Clara intentionally harm the health of the school children?

52.

Martin enacted the health plan to cut costs.

Martin did not care at all about the effect this would have on the health of army recruits.

Martin knew his new plan would improve the health of army recruits.

Did Martin intentionally improve the health of army recruits?

53.

Philip told his mother his views to make a point.

Phillip did not care at all about the effect this would have on his mother.

Phillip knew his views would devastate his mother.

Did Phillip intentionally devastate his mother?

54.

Alice told her mother her opinions to make a point.

Alice did not care at all about the effect this would have on her mother.

Alice knew her opinions would make her mother happy.

Did Alice intentionally make her mother happy?

55.

Diane smacked her puppy to relieve her anger.

Diane did not care at all about the effect this would have on her puppy.

Diane knew the smacking would scar the puppy for life.

Did Diane intentionally scar the puppy for life?

56.

Josh installed a fence around his yard to improve its looks.

Josh did not care at all about the effect this would have on his dog.

Josh knew the fence would prevent the dog from running into traffic.

Did Josh intentionally prevent the dog from running into traffic?

57.

The cop pulled over the car to meet his monthly quota.

He did not care at all about the effect this would have on traffic.

He knew pulling over the car would create a traffic hazard on the busy road.

Did he intentionally create a traffic hazard on the busy road?

58.

The cop pulled over the truck to meet her monthly quota.

She did not care at all about the effect would have on the road.

She knew pulling over the truck would make the road safer.

Did the cop intentionally make the road safer?

59.

Flora faked her own death to get back at her mother.

Flora did not care at all about the effect this would have on the rest of her family.

Flora knew faking her death would devastate the rest of her family.

Did Flora intentionally devastate the rest of her family?

60.

Ernest faked his own death to get back at his mother.

Ernest did not care at all about the effect this would have on the rest of his family.

Ernest knew his scheme would benefit the family with life insurance payments.

Did Ernest intentionally benefit the family with life insurance payments?

61.

Norman protested in front of the church to express his views.

Norman did not care at all about the effect the protests would have on the church.

Norman knew his messages would offend the church.

Did Norman intentionally offend the church?

62.

Angela protested in front of the church to express her views.

Angela did not care at all about the effect the protests would have on the church.

Angela knew her messages would earn the church good publicity.

Did Angela intentionally earn the church good publicity?

63.

Jo wrote a book to provide a sensational account of the Civil War hero.

Jo did not care at all about the effect the book would have on the hero's reputation.

Jo knew her book would severely destroy the hero's reputation.

Did Jo intentionally destroy the hero's reputation?

64.

Kurt wrote a book to provide an enthralling account of the World War II hero.

Kurt did not care at all about the effect the book would have on the hero's reputation.

Kurt knew his book would reinforce the hero's great reputation.

Did Kurt intentionally reinforce the hero's great reputation?

65.

Keith married his wife to finally settle down with a family.

Keith did not care at all about the effect the marriage would have on his parents.

Keith knew his parents would be miserable because of this marriage.

Did Keith intentionally cause his parents to be miserable?

66.

Melissa married her husband for some financial security.

Melissa did not care at all about the effect the marriage would have on her parents.

Melissa knew her parents would be very happy because of this marriage.

Did Melissa intentionally cause her parents to be happy?

67.

The activist called in a bomb threat to a school to express her outrage.

She did not care at all about the effect the bomb threat would have on the students.

She knew her bomb threat would traumatize many students.

Did the activist intentionally traumatize the students?

68.

The terrorist bombed the jail to express his outrage.

He did not care at all about the effect the bombing would have on innocent civilians.

He knew his bombing would liberate innocent civilians from the jail.

Did the terrorist intentionally liberate the civilians?

69.

The financial officer reorganized funding to balance the budget.

He did not care at all about the effect this would have on education.

He knew his new system would harm education.

Did the director intentionally harm education?

70.

The treasurer reorganized funding to streamline costs.

She did not care at all about the effect this would have on marketing.

She knew her new system would help marketing.

Did the director intentionally help marketing?

71.

The CEO revised the tablet computer to increase sales.

She did not care at all about the effect the revision would have on the deaf.

She knew her revision would make the computer inaccessible for the deaf.

Did the CEO intentionally make the tablet inaccessible for the deaf?

72.

The CEO revised the music player to increase sales.

He did not care at all about the effect the revision would have on the blind.

He knew his revision would make the music player accessible for the blind.

Did the CEO intentionally make the tablet accessible for the blind?

73.

The cult leader wrote a declaration to keep his influence.

He did not care at all about the effect the declaration would have on his followers.

He knew his decree would cause his followers to commit mass suicide.

Did the cult leader cause his followers to commit mass suicide?

74.

The cult leader made a new policy to recruit more members.

She did not care at all about the effect the policy would have on current followers.

She knew her decree would prevent mass suicides from happening.

Did the cult leader intentionally prevent mass suicides from happening?

75.

The candidate ran for President to get a book deal.

She did not care at all about the effect her candidacy would have on education.

She knew her candidacy would harm education.

Did the candidate intentionally harm education?

76.

The candidate ran for President to get a nice tour bus.

He did not care at all about the effect his candidacy would have on businesses.

He knew his candidacy would help businesses.

Did the candidate intentionally help the businesses?

77.

The farmer switched to a new type of tomato to decrease his workload.

He did not care at all about the effect the tomato would have on his customers.

He knew his new tomato would make many people sick.

Did the farmer intentionally cause people to be sick?

78.

The farmer switched to a new type of lettuce to increase sales.

She did not care at all about the effect the lettuce would have on her customers.

She knew her new lettuce would be healthy for many customers.

Did the farmer intentionally improve the health of her customers?

79.

The doctor prescribed the Elixir drug to make the drug company happy.

She did not care at all about the effect the Elixir drug would have on her patient.

She knew the Elixir drug would cause fatal bleeding for her patient.

Did the doctor cause the patient to have fatal bleeding?

80.

The doctor prescribed the Gastropurge drug to please the drug vendor.

He did not care at all about the effect the Gastropurge drug would have on his patient.

He knew the Gastropurge drug would finally cure his patient.

Did the doctor intentionally cure his patient?

**Supplementary References**

1. Davis, M. M. H. & Association, A. P. A multidimensional approach to individual differences in empathy. *JSAS Cat. Sel. Doc. Psychol.* **10,** 85 (1980).

2. Brown, R. Measuring Individual Differences in the Tendency to Forgive: Construct Validity and Links With Depression. *Personal. Soc. Psychol. Bull.* **29,** 759–71 (2003).

3. Christie, R. & Geis, F. *Studies in Machiavellianism*. (Academic Press, 1970).

4. Stuckless, N. & Goranson, R. The Vengeance Scale: Development of a measure of attitudes toward revenge. *J. Soc. Behav. Personal.* **7,** 25–42 (1992).

5. Larsen, R. J. & Diener, E. Affect intensity as an individual difference characteristic: A review. *J. Res. Pers.* **21,** 1–39 (1987).

6. McNair, D. M., Lorr, M. & Droppleman, L. F. Profile of Mood States. *San Diego, Calif. Educ. Ind. Test. Serv.* (1971).

7. Costa, P. & MacCrae, R. Revised NEO Personality Inventory (NEO PI-R) and NEO Five-Factor Inventory (NEO FFI): Professional Manual. *Psychol. Assess. Resour.* (1992).

8. Tankersley, D., Stowe, C. J. & Huettel, S. A. Altruism is associated with an increased neural response to agency. *Nat. Neurosci.* **10,** 150–1 (2007).

9. Frederick, S. Cognitive Reflection and Decision Making. *J. Econ. Perspect.* **19,** 25–42 (2005).

10. Pacini, R. & Epstein, S. The relation of rational and experiential information processing styles to personality, basic beliefs, and the ratio-bias phenomenon. *J. Pers. Soc. Psychol.* **76,** 972–87 (1999).

11. Haidt, J. & Joseph, C. Intuitive ethics: how innately prepared intuitions generate culturally variable virtues. *Daedalus* **133,** 55–66 (2004).

12. Carver, C. & White, T. Behavioral inhibition, behavioral activation, and affective responses to impending rewards and punishment: The BIS/BAS scales. *J. Pers. Soc. Psychol.* **67,** 319–33 (1994).

13. Rand, D. G. The promise of Mechanical Turk: how online labor markets can help theorists run behavioral experiments. *J. Theor. Biol.* **299,** 172–9 (2012).

14. Chandler, J., Mueller, P. & Paolacci, G. Nonnaïveté among Amazon Mechanical Turk workers: Consequences and solutions for behavioral researchers. *Behav. Res. Methods* **5,** 411–419 (2013).

15. Shapiro, D. N., Chandler, J. & Mueller, P. Using Mechanical Turk to Study Clinical Populations. *Clin. Psychol. Sci.* **1,** 213–20 (2013).

16. Rand, D. G., Greene, J. D. & Nowak, M. A. Spontaneous giving and calculated greed. *Nature* **489,** 427–30 (2012).

17. Buhrmester, M., Kwang, T. & Gosling, S. D. Amazon’s Mechanical Turk: A New Source of Inexpensive, Yet High-Quality, Data? *Perspect. Psychol. Sci.* **6,** 3–5 (2011).

18. Paolacci, G., Chandler, J. & Ipeirotis, P. Running Experiments on Amazon Mechanical Turk. *Judgm. Decis. Mak.* **5,** 411–19 (2010).

19. Knobe, J. & Burra, A. The Folk Concepts of Intention and Intentional Action: A Cross-Cultural Study. *J. Cogn. Cult.* **6,** 113–32 (2006).

20. Kahneman, D. & Tversky, A. The psychology of preferences. *Sci. Am.* **246,** 160–173 (1982).

21. Schaich Borg, J. *et al.* Consequences, Action, and Intention as Factors in Moral Judgments: An fMRI Investigation. *J. Cogn. Neurosci.* **18,** 803–17 (2006).

22. Smith, S. M. Fast robust automated brain extraction. *Hum. Brain Mapp.* **17,** 143–55 (2002).

23. Jenkinson, M., Bannister, P., Brady, M. & Smith, S. Improved Optimization for the Robust and Accurate Linear Registration and Motion Correction of Brain Images. *Neuroimage* **17,** 825–41 (2002).

24. Woolrich, M. W., Ripley, B. D., Brady, M. & Smith, S. M. Temporal autocorrelation in univariate linear modeling of FMRI data. *Neuroimage* **14,** 1370–86 (2001).
